# The metabaging cycle promotes non‐metabolic chronic diseases of ageing

**DOI:** 10.1111/cpr.13712

**Published:** 2024-07-11

**Authors:** Yeqian Cheng, Ruirui Liu, Ruiqi Rachel Wang, Kang Yu, Ji Shen, Jing Pang, Tiemei Zhang, Hong Shi, Liang Sun, Ng Shyh‐Chang

**Affiliations:** ^1^ Key Laboratory of Organ Regeneration and Reconstruction, State Key Laboratory of Stem Cell and Reproductive Biology Institute of Zoology, Chinese Academy of Sciences Beijing China; ^2^ Institute for Stem Cell and Regeneration, Chinese Academy of Sciences Beijing China; ^3^ University of Chinese Academy of Sciences Beijing China; ^4^ Beijing Institute for Stem Cell and Regenerative Medicine Beijing China; ^5^ Department of Clinical Nutrition, Department of Health Medicine Peking Union Medical College Hospital, Chinese Academy of Medical Sciences & Peking Union Medical College Beijing China; ^6^ Peking Union Medical College Hospital Beijing China; ^7^ Department of Geriatrics, Beijing Hospital, National Center of Gerontology Institute of Geriatric Medicine, Chinese Academy of Medical Sciences Beijing China; ^8^ The Key Laboratory of Geriatrics Beijing Institute of Geriatrics, Institute of Geriatric Medicine, Chinese Academy of Medical Sciences, Beijing Hospital, National Center of Gerontology of National Health Commission Beijing China; ^9^ The NHC Key laboratory of Drug Addiction Medicine Kunming Medical University Kunming China

The global trend of population ageing is intertwined with the rising incidence of metabolic diseases such as obesity and muscle atrophy, posing a formidable challenge to human health. A more profound understanding of the mechanisms linking metabolic and chronic diseases can enhance the standard of human health and contribute to achieving healthy ageing.^1^, ^2^, ^3^, ^4^ The Metabaging Cycle concept introduced by Ma and Shyh‐Chang in 2022^5^ unveiled the intricate interplay between metabolic dysregulation and inflammation in both adipose and muscle tissue, ultimately leading to the occurrence of obesity and muscle atrophy. Specifically, excesive lipids not only promote inflammation and ageing processes in adipose tissue, diminishing the secretion of beneficial adipose factors, but also triggers muscle fat infiltration and mitochondrial dysfunction. The interaction between inflammatory factors and adipose or muscle tissue further exacerbates systemic insulin resistance and chronic inflammation.

The Metabaging Cycle theory underscores the close connection between metabolic health in muscle and adipose tissue and overall well‐being, which manifests especially clearly in pathological conditions like obesity, insulin resistance and cachexia. This vicious cycle serves as a driving force for various chronic metabolic syndrome diseases and further promotes the pathogenesis of non‐metabolic chronic diseases of ageing such as neurodegenerative diseases, osteoporosis, arthritis and cancer (Figure 1).^6^, ^7^, ^8^, ^9^, ^10^ Hence, from the perspective of the Metabaging Cycle theory, disrupting this malignant cycle stands as a key strategy in preventing and treating a large variety of chronic diseases of ageing, holding significant importance in reducing the incidence risk of chronic diseases and enhancing overall health.

**FIGURE 1:**
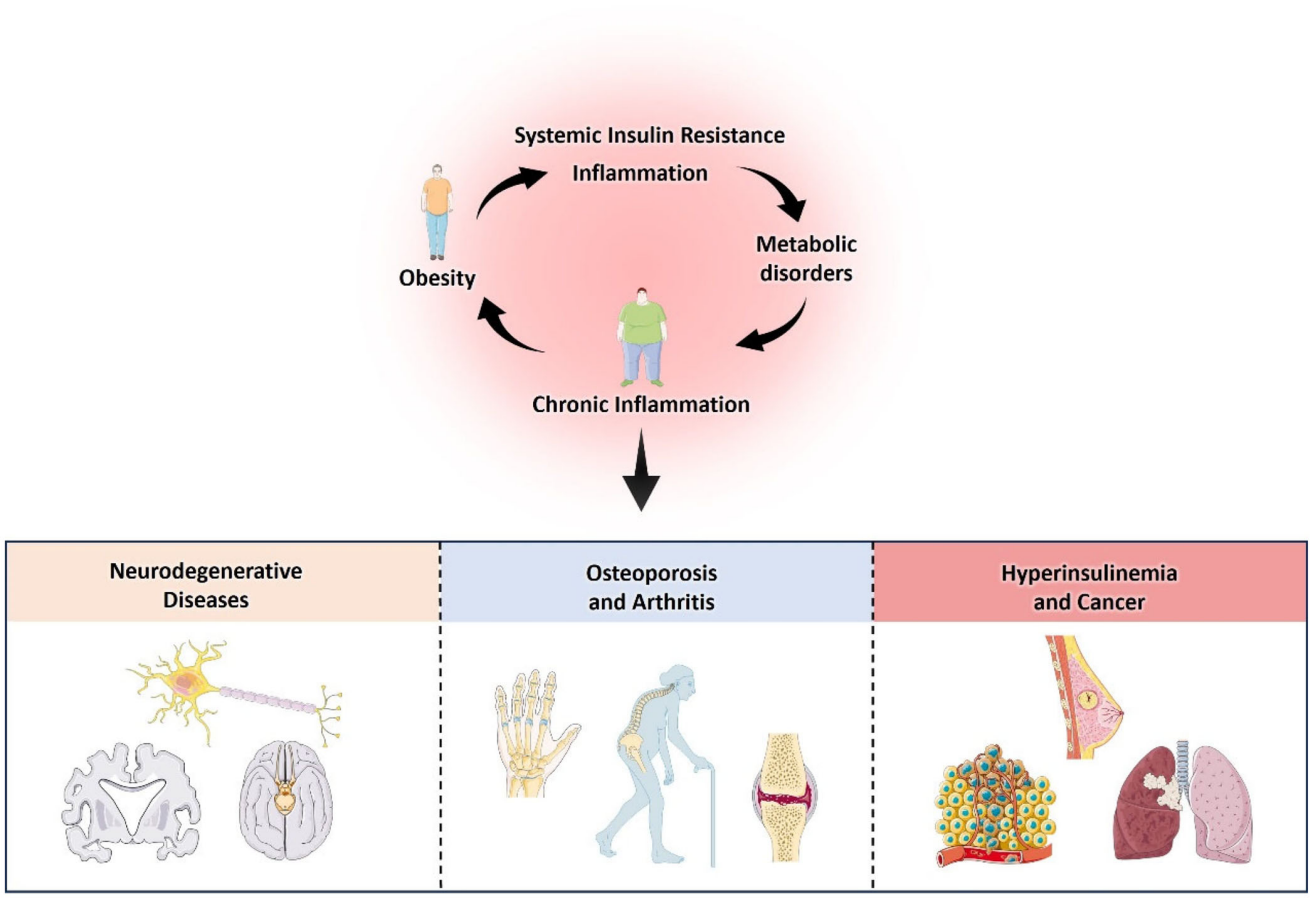
The Metabaging Cycle Promotes Non‐Metabolic Chronic Diseases of Ageing: Exploring the link between metabolic disorders, inflammation and chronic diseases like neurodegeneration, osteoporosis, arthritis and cancer. Chronic inflammation and the associated systemic insulin resistance are two major underlying causes.

In the field of neuroscience, chronic inflammatory states have been confirmed as a critical pathogenic factor.^11^ This inflammatory state, through sustained stimulation by inflammatory mediators such as cytokines and free radicals, triggers neuronal damage and neurodegenerative changes. Specifically, cytokines such as tumour necrosis factor‐alpha (TNF‐α) and interleukin‐1 beta (IL‐1β) play essential roles in the process of neuroinflammation, activating microglial cells and astrocytes to produce more inflammatory mediators, creating a vicious cycle and positive feedback loop that exacerbates neuronal damage.^12^, ^13^, ^14^ Furthermore, free radicals such as reactive oxygen species (ROS) and reactive nitrogen species (RNS) can directly damage neuronal membranes, leading to lipid peroxidation and protein oxidation, disrupting the normal function and structure of neurons.^15^ This inflammation disrupts neuronal function and structure and interferes with nerve cell metabolism and signal transduction, accelerating the development of neurodegenerative diseases.^16^ For instance, chronic inflammation can result in mitochondrial dysfunction, reducing energy supply, impacting the survival and function of nerve cells.^17^ Additionally, inflammatory mediators can interfere with signalling pathways within nerve cells, such as the NF‐κB pathway, affecting gene expression and cellular function.^18^ Moreover, inflammation can lead to the breakdown of the blood–brain barrier (BBB), making it easier for peripheral immune cells to enter the brain, exacerbating neuroinflammation.^19^ Concurrently, systemic insulin resistance and adipose tissue inflammation act on brain energy metabolism, leading to insufficient energy supply, further affecting the function of nerve cells. Insulin resistance, characterized by reduced sensitivity of target tissues to insulin, results in elevated blood sugar and triggers various metabolic disruptions. In the field of neuroscience, insulin resistance not only impacts brain energy metabolism but also influences neuronal growth, differentiation and survival through the insulin‐like growth factor 1 (IGF‐1) pathway.^20^, ^21^

Systemic insulin resistance also severely affects bone and muscle health. Insulin resistance‐induced metabolic disturbances in bones and muscles not only impair the health of the nervous system but also contribute to the development of osteoporosis and muscle atrophy. Osteoporosis is a disease in which bone integrity and strength are impaired and results from decreased bone density and microstructural deterioration, a pathophysiology that increases susceptibility to fractures. Insulin resistance, in turn, disrupts the normal balance of bone remodelling by impairing the differentiation and activity of both osteoblasts, which are responsible for bone formation and osteoclasts, involved in bone resorption. This dysregulation culminates in a net decrease in bone mass, thereby triggering osteoporosis.^22^, ^23^ Additionally, muscle atrophy refers to the reduction in muscle mass and strength, mainly manifested as decreased muscle volume and decreased muscle strength.^24^ Insulin resistance influences the energy metabolism and protein synthesis of muscle cells, resulting in decreased muscle mass and strength, thereby increasing the risk of falls and fractures.^25^ Adverse effects on joint health are also observed due to adipose tissue inflammation and muscle fat infiltration, exacerbating arthritis symptoms. Previous research has demonstrated that Lin28a expression in the skeletal muscles can lead to enhanced insulin sensitivity, reduced ectopic adiposity and maintenance of a subset of adult muscle stem cells in an embryonic‐like state, which is important for musculoskeletal regeneration and resistance to muscle atrophy.^26^, ^27^, ^28^, ^29^ Conversely, deficiency in muscle Lin28a and accumulation of *let‐7* microRNAs can lead to insulin resistance and the premature ageing or dysfunction of musculoskeletal progenitor cells. These research suggest that insulin resistance may also impact stem cell function, further exacerbating musculoskeletal health.

Adipose tissue inflammation also triggers the secretion of adipocyte‐derived factors such as tumour necrosis factor‐alpha (TNF‐α) and interleukin‐6 (IL‐6), which promote inflammatory responses, stem cell dysfunction and musculoskeletal damage. In fact, excessive inflammation‐fatty acid oxidation‐mtROS‐p38 signalling is a major contributing factor to the muscle progenitor death and severe muscle atrophy observed in cachexia, a syndrome similar to ageing‐associated sarcopenia and its reversal can ameliorate musculoskeletal atrophy.^30^, ^31^ Simultaneously, muscle fat infiltration also adversely affects normal muscle function and bone density, thereby increasing joint stress and the risk of arthritis.^32^, ^33^ The presence of obesity and insulin resistance further exacerbates these issues. Obesity increases the joint stress, resulting in cartilage wear and chondrocyte inflammation and consequently increases the risk of arthritis.

Concurrently, the intricate connection between obesity and cancer is undeniable.^34^ The tissues in the bodies of obese individuals generate inflammatory mediators, which heighten the risk of cancer development in various organs,^35^, ^36^, ^37^ including colorectal, endometrial, ovarian, breast, prostate, thyroid, oesophageal, liver, pancreatic, kidney cancer, and so forth. During insulin resistance, tissues such as muscle, fat and liver exhibit reduced sensitivity to insulin, prompting the pancreas to secrete more insulin to lower blood sugar levels, resulting in hyperinsulinemia. This elevated insulin level fosters cell proliferation and inhibits apoptosis, providing a conducive environment for cancer cell growth and metastasis, leading to malignancy.^38^, ^39^, ^40^ The exacerbation of the inflammatory response by hyperglycemia, another consequence of peripheral insulin resistance during obesity, further creates a vicious cycle that promotes tumour cell proliferation and metastasis. Moreover, hyperinsulinemia also raises the levels of insulin‐like growth factor‐1 (IGF‐1), which not only stimulates cell proliferation but also inhibits apoptosis and senescence, further fueling the growth and spread of cancer cells.^41^, ^42^ Muscle fat infiltration may also lead to muscle atrophy, compromising patients' protein stores and adaptive immune system, subsequently impacting chemotherapy and radiotherapy options, treatment outcomes and quality of life. In summary, factors such as obesity, insulin resistance and hyperglycemia intensify proliferative signalling, oxidative stress and inflammatory responses within the body, causing DNA damage, selecting for cancerous genetic mutations, promoting the growth and spread of cancer cells, thus rendering them more malignant and invasive.

In summary, metabolic disorders like obesity, insulin resistance and muscle atrophy are significant risk factors for various chronic diseases. These conditions interact through inflammatory factors and metabolic disruptions, affecting the insulin sensitivity and muscle function, thereby increasing the risk of neurodegenerative diseases, osteoporosis, arthritis, cancer and other chronic illnesses. Nevertheless, it is important to acknowledge the limitations of the current understanding of the Metabaging Cycle and its implications for chronic disease development and progression. Despite the growing recognition of the intricate relationship between metabolic disorders and chronic diseases, the exact mechanisms and pathways by which these elements influence the onset and progression of diseases remain incompletely elucidated. Future research should focus on addressing these gaps in knowledge. Future research and medical practices should also focus on comprehensive interventions, such as improving dietary habits, increasing physical activity,^43^, ^44^ managing weight and pharmacological therapies,^45^, ^46^, ^47^ to prevent and treat these diseases. Through these efforts, we can effectively mitigate the rising trend of global chronic diseases, enhance human health and contribute to achieving ageing.

## AUTHOR CONTRIBUTIONS

YC, RL, RRW, KY, JS, JP, TZ, HS, LS and NS‐C designed and wrote the manuscript.

## CONFLICT OF INTEREST STATEMENT

The authors declare that the research was conducted in the absence of any commercial or financial relationships that could be construed as a potential conflict of interest. Ng Shyh‐Chang is an Editorial Board member of Cell Proliferation and a coauthor of this article. He was excluded from the editorial decision making related to the acceptance of this article for publication in the journal.
